# Supplementary material for: Systematic clustering algorithm for chromatin accessibility data and its application to hematopoietic cells
Source: PLoS Comput Biol. 2020 Nov 30;16(11):e1008422. doi: 10.1371/journal.pcbi.1008422 (PMC7728210; doi:10.1371/journal.pcbi.1008422)
Supplement: S1 Appendix — (PDF) [file pcbi.1008422.s001.pdf]

Additional details of sequencing analysis

Azusa Tanaka[1,2*◕], Yasuhiro Ishitsuka[3,4*◕], Hiroki Ohta[3,5*◕], Akihiro Fujimoto[1], Jun-ichirou Yasunaga[2,6], Masao Matsuoka[2,6]

1 Department of Human Genetics, Graduate School of Medicine, The University of Tokyo, Tokyo, Japan
2 Laboratory of Virus Control, Institute for Frontier Life and Medical Sciences, Kyoto University, Kyoto, Japan
3 Center for Science Adventure and Collaborative Research Advancement, Graduate School of Science, Kyoto University, Kyoto, Japan
4 Department of Mathematics, Graduate School of Science, Kyoto University, Kyoto, Japan
5 Department of Physics, Graduate School of Science, Kyoto University, Kyoto, Japan
6 Department of Hematology, Rheumatology and Infectious Disease, Faculty of Life Sciences, Kumamoto University, Kumamoto, Japan

◕These authors contributed equally to this work.
* a-tanaka@m.u-tokyo.ac.jp (AT), yasu-ishi@math.kyoto-u.ac.jp (YI), ohta.hiroki.6c@kyoto-u.ac.jp (HO)

Abstract

This appendix explains a rather conventional way to preprocess ATAC-seq in detail.

1 ATAC-seq: Analysis for open chromatin regions based on Tn5-transposase

Throughout this appendix, we used hg19 as the human reference sequence. It consists of 24 groups of symbol sequences, which corresponds to chromosomes labeled as $\mathbb{X} := \{1, 2, \ldots, 22, \mathrm{X}, \mathrm{Y}\}$. The underlying structure of a chromosome is a long chain of DNA and the DNA is represented as a sequence of elements in set

$$\mathbb{D} := \{\mathrm{A}, \mathrm{T}, \mathrm{G}, \mathrm{C}\},$$

where each symbol corresponds to the nucleotides adenine (A), thymine (T), guanine (G), and cytosine (C).

For the γ-th chromosome ($\gamma \in \mathbb{X}$), the length of the corresponding DNA sequence is written as L_γ, where $5.0 \times 10^7 \leq L_\gamma \leq 2.5 \times 10^8$ and the total length is $L = \sum_{\gamma=1}^{22} L_\gamma + L_\mathrm{X} + L_\mathrm{Y} \sim 3.1 \times 10^9$. To position the x-th base pair in the γ-th chromosome, we setchromatin

$$b_x^\gamma \in \mathbb{D},$$

with $1 \leq x \leq L_\gamma$. In this paper, for a set $\mathbb{SET}$, we write the number of elements in $\mathbb{SET}$ as $|\mathbb{SET}|$. For example, we have $|\,\mathbb{D}\,| = 4$ and $|\mathbb{X}| = 24$.

Chromatin is a complex of DNA and associated proteins such as histones. A chromatin has “open” regions, around which the density of the DNA and the associated proteins are rather low and also “closed” regions, around which the opposite situation

happens. Gene expressions are largely regulated by the interactions between DNA and transcription factors depending on the open and closed regions. The analysis of open/closed chromatin regions is necessary for the understanding of cell differentiation and phenotype [1, 2].

ATAC-seq was developed for the genome-wide detection of open chromatin regions. One of the features of ATAC-seq is that it uses Tn5 transposase. At a certain proper condition, Tn5-transposase mainly cut DNA in open chromatin regions and the sequences of those DNA fragments are obtained by sequencers [3]. ATAC-seq has several advantages compared to the other epigenomic sequencing methods [4]. For example, to analyze open chromatin regions, DNase-seq needs about 10^7–10^8 cells and takes 4–5 days to obtain the data. ATAC-seq, on the other hand, requires only about 10^3–10^4 cells and takes half a day.

2 Reads

As briefly reviewed above, one Tn5-transposase cuts and splits DNA into two parts or fragments. If there are two Tn5-transposases, two locations of DNA are cut to make three fragments.

Thus, we can view *fragment* f as a subsequence of a DNA sequence consisting of successive symbols in $\mathbb{D}$. Since we refer to the same DNA sequence of the human genome in this study, fragment f can be also represented by three coordinates: the the chromosome number $\gamma \in \mathbb{X}$, the start position $s = s(f)$, and the end position $e = e(f)$, where $1 \leq s \leq e \leq L_\gamma$. In other words, f is a sequence $(b_s^\gamma, b_{s+1}^\gamma, \ldots, b_e^\gamma)$, that can be expressed as $f = (\gamma, s, e)$.

A *sample* is, in our settings, a product generated by a certain experimental procedure through ATAC-seq library preparation from a set of cells [3].

The input of a sequencer is the set of the obtained fragments $\{f_i\}_{i=1}^{N_\mathrm{f}}$, where a fragment f_i is (γ_i, s_i, e_i), its length $L(f_i)$ is equal to $e_i - s_i + 1$, and the number of fragments is denoted as N_f. A sequencer with "paired-end sequencing" outputs a DNA sequence of the two edges of a fragment as two reads $(\mathbf{R}_i^s, \mathbf{R}_i^e)$ where

$$\mathbf{R}_i^s := (\mathrm{R}_j)_{j=1}^{\ell_i},\ \mathbf{R}_i^e := (\mathrm{R}'_j)_{j=1}^{\ell_i} \text{ for } \mathrm{R}_j, \mathrm{R}'_j \in \mathbb{D},$$

meaning that each length of the two reads $(\mathbf{R}_i^s, \mathbf{R}_i^e)$ is ℓ_i.

We consider *read* as a sequence of four symbols in $\mathbb{D}$ of length less than or equal to ℓ_0, where ℓ_0 can be changed as a parameter controlled by the sequencer. Note that for the case of "single-end sequencing", where one gets only a read from one edge, we obtain read $\mathbf{R}_i = \{\mathrm{R}_j\}_{j=1}^{\ell_i}$. In the end, we obtain the data of reads $\mathbf{R} := \{\mathbf{R}_i\}_{i=1}^{N'_\mathrm{r}}$ where the number of reads is denoted as N'_r. Note that in the case of "paired-end sequencing", one may regard both $\mathbf{R}_i^s$ and $\mathbf{R}_i^e$ as $\mathbf{R}_i$. This is the starting point of our analysis because sequencers do not directly give the actual values of f_i.

Summarizing the relationship between fragments and reads, let us assume that all reads are obtained from "paired-end sequencing" and that the sample preparation and the sequencer output are "ideal" as follows. If we denote fragment f_i as sequence $(b_{s_i}^{\gamma_i}, b_{s_i+1}^{\gamma_i}, \ldots, b_{e_i}^{\gamma_i})$, then the beginning read $\mathbf{R}_i^s$ and the terminal read $\mathbf{R}_i^e$ corresponding to f_i are

$$\mathbf{R}_i^s = \begin{cases} (b_{s_i}^{\gamma_i}, b_{s_i+1}^{\gamma_i}, \ldots, b_{s_i+\ell_0-1}^{\gamma_i}) & \text{for } \ell_0 \leq L(f_i), \\ (b_{s_i}^{\gamma_i}, b_{s_i+1}^{\gamma_i}, \ldots, b_{e_i}^{\gamma_i}) & \text{for } \ell_0 > L(f_i), \end{cases}$$

$$\mathbf{R}_i^e = \begin{cases} (b_{e_i-\ell_0+1}^{\gamma_i}, b_{e_i-\ell_0+2}^{\gamma_i}, \ldots, b_{e_i}^{\gamma_i}) & \text{for } \ell_0 \leq L(f_i), \\ (b_{s_i}^{\gamma_i}, b_{s_i+1}^{\gamma_i}, \ldots, b_{e_i}^{\gamma_i}) & \text{for } \ell_0 > L(f_i). \end{cases}$$

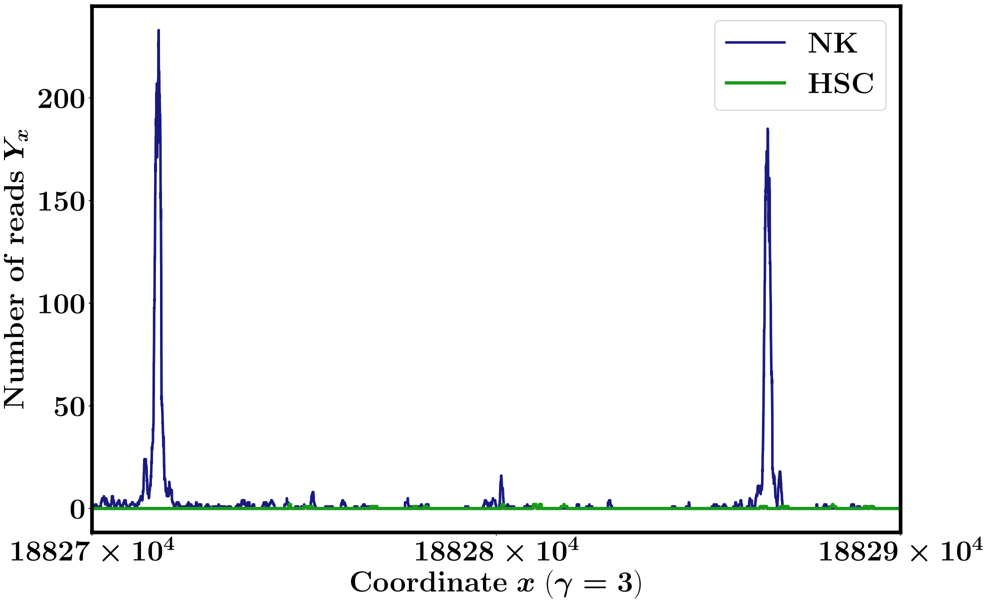

Fig S1. The number of reads vs genomic position. The number $Y_x = Y_{\gamma,x}$ of reads in the ATAC-seq data (vertical axis) vs position x in the DNA sequence (horizontal axis), where x starts from 18827×10^4 and ends at 18829×10^4, and chromosome number $\gamma = 3$.

In other words, if the length $L(f_i)$ of fragment f_i is greater than or equal to ℓ_0, the beginning read $\mathbf{R}_i^s$ is the direct inference of the first ℓ_0 symbols of the fragment f_i. The condition for terminal read $\mathbf{R}_i^e$ is similar. If length $L(f_i)$ is less than ℓ_0, we directly infer $\mathbf{R}_i^s = \mathbf{R}_i^e = f_i$ as a sequence of four symbols, where we see that the two reads have the same length.

However, the situation above is "ideal" and there are unexpected errors that stochastically flip symbols in the ideal situation. Thus, we need to infer the information of fragments in a statistical manner. Note that this inference can be straightforwardly applied to the case of "single-end sequencing".

3 Alignment of reads onto the reference genome

Hereafter, for simplicity, we consider single-ended reads $\mathbf{R} = \{\mathbf{R}_i\}_{i=1}^{N'_\mathrm{r}}$ because similar processes can be done for paired-end reads. We perform mapping of the reads data $\mathbf{R}$ from a sequencer onto the DNA sequence.

We use the BWA-MEM algorithm of the software *BWA* (v0.7.16a) with no options. This algorithm aligns each read onto the hg19 reference sequence $(b_x^\gamma)_{\gamma \in \mathbb{X}, 1 \leq x \leq L_\gamma}$ and gives an estimate of the quality of the alignment (for details, see [5] and references therein). Then we obtain the following data:

- Chromosome number $\widehat{\gamma}(\mathbf{R}_i) \in \mathbb{X} \cup \{\mathrm{U}\}$ with the start position $\widehat{s}(\mathbf{R}_i)$ and the end position $\widehat{e}(\mathbf{R}_i)$ of read $\mathbf{R}_i$ mapped onto the DNA sequence, where $1 \leq \widehat{s}(\mathbf{R}_i) \leq \widehat{e}(\mathbf{R}_i) \leq L_{\widehat{\gamma}(\mathbf{R}_i)}$. Note that U is a set of unplaced sequences in any elements in $\mathbb{X}$. Hereafter, $\mathbb{X}$ includes U with $L_\mathrm{U} \simeq 3.7 \times 10^6$.

- The mapping quality score $\mathrm{MQ}(\mathbf{R}_i) \geq 0$ of read $\mathbf{R}_i$ calculated by using the Phred quality score.

Therefore, $(\widehat{\gamma}(\mathbf{R}_i), \widehat{s}(\mathbf{R}_i), \widehat{e}(\mathbf{R}_i))$ infers the coordinates $(\gamma_i, s_i, e_i) = (\widehat{\gamma}(\mathbf{R}_i), \widehat{s}(\mathbf{R}_i), \widehat{e}(\mathbf{R}_i))$ of read $\mathbf{R}_i$ onto the DNA sequence. For $\mathbf{R}_i$, we define $\hat{\mathbf{T}}(\mathbf{R}_i)$ as

$$\hat{\mathbf{T}}(\mathbf{R}_i) := (\widehat{\gamma}(\mathbf{R}_i), \widehat{s}(\mathbf{R}_i), \widehat{e}(\mathbf{R}_i), \mathrm{MQ}(\mathbf{R}_i)).$$

To select reliable data with $\hat{\mathbf{T}}(\mathbf{R}_i)$, we preprocess the outputs obtained above as follows:

1. In order to reduce duplicated reads, which could be produced artificially in the sequence sample preparation, we apply the command MarkDuplicates in PICARD software (v1.119) (http://broadinstitute.github.io/picard/) with the REMOVE_DUPLICATE option.

2. Then we cut off reads with a mapping quality score $\mathrm{MQ}(\mathbf{R}_i)$ less than 30. We used *samtools* for this purpose [6].

After processes (1) and (2), we obtain

$$\hat{\mathbf{P}}(\mathbf{R}') := \{(\widehat{\gamma}(\mathbf{R}'_i), \widehat{s}(\mathbf{R}'_i), \widehat{e}(\mathbf{R}'_i))\}_{i=1}^{N_\mathrm{r}},$$

where ℓ'_i is the length of $\mathbf{R}'_i$ and N_r denotes the number of reads after preprocessing. $\{\mathbf{R}'_i\}_{i=1}^{N_\mathrm{r}}$ can be straightforwardly determined by $\{\mathbf{R}_i\}_{i=1}^{N'_\mathrm{r}}$. This is part of the information obtained by the preprocessing. Note that $\mathrm{MQ}(\mathbf{R}'_i) \geq 30$ holds for any i with $1 \leq i \leq N_\mathrm{r}$ and there are no duplicated pairs in $\hat{\mathbf{P}}$. For simplicity, hereafter, we sometimes express $\hat{\mathbf{P}}(\mathbf{R})$ as $\hat{\mathbf{P}}$. We use similar abbreviations for other symbols.

4 Pilings of reads

From the data $\hat{\mathbf{P}}$, we can calculate how many reads are on position (γ, x) in the DNA sequence. We consider the set of reads located on position (γ, x) symbolically by defining

$$\mathbb{Y}_{\gamma,x}(\hat{\mathbf{P}}(\mathbf{R})) := \{1 \leq i \leq N_\mathrm{r} \mid \widehat{\gamma}(\mathbf{R}_i) = \gamma \text{ and } (\ \widehat{s}(\mathbf{R}_i) \leq x \leq \widehat{e}(\mathbf{R}_i))\}.$$

For two samples in reads data $\mathbf{R}$ obtained from SRA (SRR2920495.sra and SRR2920466.sra), we show $Y_{\gamma,x} := |\mathbb{Y}_{\gamma,x}|$, which is the number of reads on each position (γ, x) in the DNA sequence in Fig S1. In this study, we used reads data $\mathbf{R}$ from the Gene Expression Omnibus (GEO) with accession number GSE74912 as the initial input of the analysis.

References

1. Bradbury J. Human Epigenome Project—Up and Running. *PLoS Biology.* 2003; 1(3):e82.

2. Gasper-Maia A, Alajem A, Meshorer E, Ramalho-Santos M. Open chromatin in pluripotency and reprogramming. *Nature Reviews Molecular Cell Biology.* 2011; 12(1):36-47.

3. Buenrostro JD, Giresi PG, Zaba LC, Chang HY, Greenleaf WJ. Transposition of native chromatin for fast and sensitive epigenomic profiling of open chromatin, DNA-binding proteins and nucleosome position. *Nature Methods.* 2013; 10(12):1213–1218.

4. Meyer CA and Liu XS. Identifying and mitigating bias in next-generation sequencing methods for chromatin biology. *Nature Reviews Genetics*. 2014; 15(11):709–721.

5. Li H. (2013). Aligning sequence reads, clone sequences and assembly contigs with BWA-MEM. *arXiv:1303.3997*. [Preprint]. 2013 Available from: `https://arxiv.org/abs/1303.3997?upload=1`

6. Li H, Handsaker B, Wysoker A, Fennell T, Ruan J, Homer N, et al. The Sequence Alignment/Map format and SAMtools. *Bioinformatics*. 2009; 25(16):2078–2079.
