# Supplementary material for: Systematic clustering algorithm for chromatin accessibility data and its application to hematopoietic cells
Source: PLoS Comput Biol. 2020 Nov 30;16(11):e1008422. doi: 10.1371/journal.pcbi.1008422 (PMC7728210; doi:10.1371/journal.pcbi.1008422)
Supplement: Attachment — Submitted filename: 103118_1_rebuttal_1719150_q89rkr.pdf [file pcbi.1008422.s002.pdf]

Dear reviewers,

Thank you for the thoughtful and constructive feedback regarding our manuscript.
Following the comments, we have revised our manuscript extensively, which we submit with this file.

Below we reply to each of the comments individually. Beforehand, we provide a summary of the major changes.

Thank you once again for your consideration of our paper.

Sincerely,

Azusa Tanaka, Yasuhiro Ishitsuka, Hiroki Ohta, Akihiro Fujimoto, Jun-ichirou Yasunaga, and Masao Matsuoka

****

Major changes:

1. Improvement of the hierarchical clustering:
We performed wider parameter searching for different clustering methods such as Ward's method. We found that the penalty of the clustering dendrogram obtained by Ward's method with M_cut =64000 is 18, which is better than the 36 obtained by UPGMA with M_cut=12000 in the original version.

2. Regarding the comparison of our algorithm with previous work [Corces et al., Nature 2016], we tried to compare the best dendrogram of our algorithm with Corces, however, we could not make a full comparison due to the following.

Corces computed a cluster purity of a dendrogram. The cluster purity can be computed after a set of samples are divided into 13 subsets (clusters), where 13 is the number of types of hematopoietic cells analyzed. However, Corces does not define the clusters explicitly.

One possible way to build clusters is to find a height below which there are 13 branches of the dendrogram. A cluster purity of 90.9% (written in Figure 2f of Corces) can be achieved when in total 70 samples out of the 77 samples are correctly assigned for each cluster. However, looking carefully at the dendrogram of Figure 2f in Corces, we see that the height where one Ery sample merges another Ery sample is generally higher than the samples of other types. Thus, if we adopt the "height-cutting method" as the way to form clusters, the number of incorrectly assigned samples is greater than 7 and the cluster purity is smaller than 90.9%.
One other way to achieve 90.9% cluster purity is to optimize the clusters of the dendrogram. With this strategy, our best clustering achieves cluster purity = 92.2%, which means that in total 71 samples out of 77 samples are correctly assigned.

We also tried to reproduce the dendrogram shown in Figure 2f of Corces by using the reads count data obtained from GSE74912_ATACseq_All_Counts.txt by Ward's method. Using this dendrogram, we computed the cluster purity by using the "height-cutting" and "optimized" methods, acquiring 81.8% (14 mismatches) and 89.6% (8 mismatches), respectively.

We also calculated the “penalty score” of the dendrogram by Corces's method with GSE74912_ATACseq_All_Counts.txt. When we applied Ward's method, the penalty score was 27, which was better than the penalty of other methods such as UPGMA, but worse than the 18 obtained from our optimal condition with M_cut =64000.

There are two possible reasons that prevented us from reproducing the 90.0% cluster purity in Corces. (i) The manner of building the clusters in Corces was neither "height-cutting" nor "optimized". (ii) The dendrogram we reproduced from GSE74912_ATACseq_All_Counts.txt is different from the one in Figure 2f in Corces.
For case (i), again, because the manner with which Corces. formed the clusters is not written explicitly, we could not trace their analysis further.
For case (ii), the dendrogram in figure 2f is not visible, which prevented precise comparison.

In conclusion, we tried to compare the dendrogram obtained by our developed algorithm with the dendrogram shown in Figure 2f of Corces, but we could not find any definite evidence to conclude which is better because of the reasons mentioned above.

However, we can still conclude reasonably that our best dendrogram is no worse than the dendrogram in Figure 2f of Corces. Moreover, because the cluster purity by our "optimized" method (92.2%) is better than theirs (90.9%), we could even argue our method is better.
In addition, we considered other aspects such as the computational cost and applicability, which are explained below.

3. Superiority of our computational cost
In the section "Computational cost of algorithm", we explicitly show the computational cost of our algorithm and the algorithm in Corces. In conclusion, our method is extremely faster when one new sample not yet analyzed is added to the set of samples already analyzed.

4. Application of our developed algorithm to other leukemia samples such as chronic lymphocytic leukemia (CLL) and adult T-cell leukemia (ATL):
In the original version of our manuscript, we analyzed only the AML samples studied in Corces. In the revised version, we also analyzed samples of CLL from Rendeiro et al. (Nat Commun 2020) and our original ATL samples.
When we analyzed the AML samples, we found constant differences between the two studies for LSCs. Most LSC samples were closest to LMPP in our algorithm, whereas in Corces, the closest normal cell type was mainly GMP. A recent study [Chung et al., 2017] revealed that CD99-positive cells are almost entirely composed of LMPP-like cells, thus, CD99-positive LSCs can be presumed to be LMPP-like LSCs in our study. These observations suggest that our characterization is more likely to infer the cell phenotype with high accuracy in comparison to Corces.
We further applied our method on CLL samples and found that our characterization of CLL coincides well with the characteristics of its cell surface markers. Also, we analyzed ATL and found that ATL samples are categorized into two classes, CD4+T-like ATL and Mono-like ATL, at the chromatin level. Although we do not have enough samples to make any definite conclusions, it seems that these two classes correspond to the subclasses of the ATL patients, acute type ATL and chronic-type ATL, respectively. For details, please refer to the main text of our manuscript. The ATAC-seq and RNA-seq data of ATL patients will be available soon. We are applying to submit our data to the DDBJ Sequence Read Archive (DRA) and DRA number will be announced as soon as we get it.

Reviewer #1 (Evidence, reproducibility and clarity (Required)):

Summary:

The manuscript presents a method for clustering and classification of ATAC-seq datasets and an application of the method to a dataset of 77 ATAC-seq samples from Corces, et. al, 2016, which are derived from healthy subjects and leukemia patients. In short, the method consists of 1) for each sample, binarize the genome into present and absent calls based on the output of the MACS2 peak caller; 2) apply UPGMA clustering with Hamming distance as distance metric on all healthy samples; 3) perform a grid search to find the optimal MACS2 p-value threshold and the number of top most significant peaks (M_cut) that result in the maximum cluster purity based on the known cell type of each healthy sample. Finally, the method uses the optimal number of peaks and the optimal MACS2 p-value threshold to derive peaks in a set of samples coming from leukemia patients and then find the closest healthy cell type for each sample. The authors also conclude that ATAC-seq signal at genes encoding cell surface markers is not relevant for correct clustering. Overall, the current manuscript does not present convincing evidence that the proposed method is better or more principled than already used procedures like the one in Corces, et. al. The conclusion about cell surface markers is also not well supported by the presented results. In addition, the manuscript would benefit from application of the method on more datasets in order to show that the method can be used more broadly to gain insights outside of the biological setting in the Corces, et al. study. The authors can strengthen the conclusions in the manuscript and improve the methodology by addressing the following points:

Reviewer #1 (Significance (Required)):

The manuscript presents an application of clustering with Hamming distance to ATAC-seq samples. However, variations of such clustering procedures are routinely applied in epigenomics studies (e.g. Corces, et. al, Fullard, Hauberg et al. Genome Res 2018, Wang, Zibetti et al Nat Comm 2019, Liu, Peters et al 2019 Sci Data). The current manuscript makes it difficult to evaluate the conceptual, technical or clinical significance or novelty of the proposed method, because it presents an application of the method to only one previously published dataset with limited insights into the underlying biology or clinical relevance. Furthermore, the comparison between the classification of the proposed method and the method from the original Corces, et. al paper is difficult to interpret, because the two methods are not rigorously evaluated against each other or

simpler baselines and against orthogonal datasets (e.g. gene expression, transcription factor binding and others) by computing appropriate statistics to assess significance. Therefore, the applicability of the current method both within and outside of the dataset from Corces, et al. seems questionable. In addition, the authors do not provide source code which limits the usability of their method and reproducibility of their results.

Audience: Provided that the outlined issues are properly addressed, such method could be of interest to the clinical and computational epigenetics community in the context of classifying samples from diseased patients.

My field of expertise: bioinformatics, epigenetics, genomics, medical genetics, computer science, data science

****Major comments:****

Comment A-1)

One conceptual problem with the method is that optimizing both M_cut and the p-value threshold for MACS2 is redundant, because one can argue that choosing a value for M_cut is equivalent to setting the p-value threshold to the p-value of the M_cut'th peak after sorting peaks by significance. Also, running MACS2 with multiple different p-value thresholds on each sample can be time consuming even for moderate number of samples. Therefore, it is not clear why the method should optimize both of these parameters. This problem can be resolved by avoiding third-party peak callers all together and instead modeling directly the read counts in fixed size genomic intervals (e.g. every 1bp, 50bp or 200bp etc) across the whole genome.

Reply A-1)

We thank the referee for the comment. The reviewer's point that optimizing both M_cut and the p-value threshold, p_G, is redundant is possiblly based on the assumption that each peak region detected by the MACS2 algorithm does not depend on p_G.

As we showed in Fig. 1, one peak region with $p\_G=10^{-2}$ becomes two peaks if $p\_G=10^{-4}$, and the start and end points of the peak regions could also depend on p_G. That is, each peak region could depend on p_G. Only after fixing a set of peaks depending on p_G did we consider the number of peaks by setting M_cut. Thus, our optimization for both M_cut and p_G is not redundant.

We found that $p\_G=10^{-2}$ and $M\_cut = 64000$ gave the smallest penalty score, which means this combination is the best in our searching resolution of the optimization. Thus, one can use $p\_G=10^{-2}$ for the analysis of leukemic cells and does not need to re-run MACS2 with different values of p_G at multiple times.

Comment A-2)

The section "Irrelevance to genomic regions corresponding to cell surface marker" does not present enough evidence to justify the conclusion that cell surface markers are, in general, irrelevant for clustering blood cell types based on ATAC-seq data. The rationale behind the methodology in this section is also very questionable. In particular, why is computing the ratio of strong to weak peaks overlapping the CD38 gene indicative of whether this gene matters for the clustering pattern? The ratio can be confounded by the total number of peaks in each sample, because the denominator is proportional to the total number of peaks. For example, the ratio will be high just because there are very few weak peaks in the whole sample and, conversely, the ratio will be very low for a noisy ATAC-seq sample with lots of weak peaks. Instead of comparing these ratios, to show that clustering does not depend CD38 and other cell surface markers, the authors can exclude all peaks overlapping CD38 and other known cell surface markers, then recompute the clustering and check if the clustering pattern changes compared to the original. The authors should also conduct the reverse experiment, which is to run their clustering method by using only peaks that overlap genes of known cell surface markers and see if this produces a random clustering pattern. To make a general claim about cell surface markers, this should be done by using _all_ relevant cell surface markers and not just CD38! The authors should also estimate the statistical significance of the differences between these clustering patterns given the number of samples. Also, the authors should present results on all available cell types and not just the three cell types chosen in this section. Finally, in addition to all of the above the authors can show whether CD38 and other cell surface markers have differential ATAC-seq signal at the promoter and at potential nearby enhancers across all cell types.

Reply A-2)

We totally agree with the referee's comments. However, it takes a substantially long time to answer each concern, which is why we prefer to answer this comment in a future study. Moreover, the answers to those questions do not affect the main objective of our current work. Thus, we

have removed this section from the revised manuscript in order to avoid any confusions about the core of our work.

Comment A-3)

In "Relevance to enhancer regions", the authors show that ATAC-seq peaks have bias towards promoter regions. It is not clear, however, how these results relate to evaluating the performance of the algorithm and how they validate, if at all, the value for M_cut=12,000 that was found by the algorithm.

Reply A-3)

For our reply to this comment, we ask the referee to see Major Changes #1 above.

Briefly, we updated our results by extending our optimization parameter space with Ward's method. Thereby, we found that the penalty score obtained from a combination of M_cut =64000 and Ward's method is much better than that from a combination of M_cut=12000 and UPGMA, which was used in the original version of our manuscript.

As the referee pointed out, we did not fully investigate the relevance of enhancer regions, although the functional annotations shown in Figure 10 showed that M_cut=64000 is close to the point where the contribution of enhancers becomes smaller than the contribution of quiescent regions. Since this issue does not influence the major results of the paper, we changed the title of this section to "Functional annotation of peaks depending on the rank", in which we characterize each peak by the functional annotation without emphasizing "the relevance to enhancers".

Comment A-4)

The "Robustness of the clustering" states that clustering is robust to the choice of clustering method (UPGMA, Ward, WPGMA etc), but the authors do not provide any figures or tables with results that support these conclusions.

Reply A-4)

In accordance with the referee’s comment, in the revised version, we show the penalty score as a function of M_cut for both UPGMA and Ward's method in Figures 12 and 9, respectively. For UPGMC, WPGMA and WPGMC, we list only the best penalty in our searching resolution, because these three methods generally produce worse penalty scores than UPGMA or Ward's method.

Comment A-5)

In "Robustness of the clustering", the authors conclude that their clustering method can be further improved by down-sampling the input reads with probability r and that r can be treated as parameter for optimization. However, as it is, this conclusion does not seem to be well supported by the presented results, because the difference in the performance appears to be miniscule and may likely be explained by random fluctuations caused by the down-sampling procedure. Therefore, the authors should show the statistical significance between the non-downsampled and down-sampled runs on a large enough sample to justify this conclusion. Finally, if the difference is indeed significant, the authors should explain why discarding real data leads to better results, which seems counter-intuitive at first glance.

Reply A-5)

We appreciate the referee's comment. For this issue, as mentioned in Major Changes #1 at the top of this letter, we updated our results by extending our optimization parameter space with Ward's method. The penalty score in combination with M_cut =64000 and Ward's method was much better than that of M_cut=12000 and UPGMA, which we used in the original analysis.

Then we analyzed how randomly-reduced reads data affect the penalty score. Our updated results showed that removing reads at probability r did not improve the clustering results. The results support the penalty with M_cut=64000 and r=0 as best and robust against small changes in data size.

We have revised the section "Robustness of the clustering" accordingly.

Comment A-6)

The interpretation of the results in "Application to Leukemic cells" is difficult. The authors show that their method and the method used in Corces, et. al both produce very similar clustering and classification patterns, but it is not clear which one is better and why should someone prefer the authors' method over the one used in Corces, et al? To support their case, the authors can investigate, for example, whether their classification method produces results that are more consistent with gene expression or other differences between the samples. The authors also state that their method can rank all cell types by how close they are to a particular leukemia sample. However, it is unclear how this ranking would compare to a simpler baseline such as computing average correlation between the signal over a joint set of peaks in the new sample and each group

of cells (similarly to Corces, et al) or even just picking the healthy sample that correlates best based on the raw ATAC-seq signal across the whole genome. The authors can investigate how their method compare to these or other simple baseline methods. This section would also benefit from showcasing more explicitly the clinical or biological utility of correctly classifying leukemia samples and why it is of interest to identify the second, third and so on closest healthy sample.

Reply A-6)

We agree that we did not show clearly why our method is preferred. However, some important information is absent in Corces, preventing an exhaustive comparison (please see Major Changes #2 above). Another big question is whether gene expressions are better characterized in terms of cell types. For this issue, we do not have convincing ideas unfortunately. Thus, in the revised version, instead of investigating RNA-seq data in detail, which remains a challenging topic for us in the future, we applied our methods to other kinds of leukemia including CLL and ATL along with AML. This result is described in Major Changes #4 and the section "Application to leukemic cells" in the main text.

From the viewpoint of clinical and biological utility, we found that our characterization of CLL coincides well with the characteristics of its cell surface markers. For ATL, we found that ATL samples are categorized into two classes, CD4+T-like ATL and Mono-like ATL, at the chromatin level. Although we do not have enough samples to make any definite conclusions, it seems that these two classes correspond to the subclasses of ATL patients, acute type ATL and chronic-type ATL, respectively. We have added this information to the revised manuscript.

Future study is needed to validate the clinical and biological utility. At the moment, we hypothesize that the second and the third closest types could be quantities that improve the inference.

Comment A-7)

The authors should provide all code (ideally as a python or R package) in order for others to be able to use their method and reproduce their results.

Reply A-7)

As mentioned by the referee, we provide the code in https://github.com/tanakanishi/findclosest : it requires only peak position data as input and provides the clustering dendrogram and the closest

cell type of the given sample as output. By this code, the reader can analyze a set of peak regions from one sample to see which type of hematopoietic cell is closer to the sample.

****Minor comments:****

Comment B-1)

The readability of the main text can be improved by removing most mathematical notations and moving them to the supplement. It would be better if the main text describes in words what is being done and avoids excessive use of mathematical notation.

Reply B-1)

One of the main objectives is to avoid ambiguous statements about our work. For that reason, we prefer to keep the present style of the paper.

Comment B-2)

At the end of the "Application to Leukemic cells" section, the authors state that their method is simple, but reading through the methods section makes it clear that the method requires re-running MACS2 multiple times on each sample in order to perform a grid search over the two parameters of the method, and a priori knowledge of the cell types in order to derive the optimal clustering. The authors should justify better why this procedure is simpler than running standard hierarchical clustering on a jointly defined set of peaks.

Reply B-2)

We apologize, as our writing has misled the referee.

If one is not careful about the dependence of peak regions on the p-value threshold, p_G, as mentioned in Reply A-1, one can use the default setting of the MACS2 algorithm, where q-value is set to 0.05. However, we were more careful about the dependence of the peaks on p_G and found $p\_G=10^{-2}$ to be the best for our searching resolution of the optimization. We applied this p_G value to leukemic cells. Thus, we do not need to re-run MACS2 with different values of p_G at multiple times for the analysis of a leukemic sample.

As explained in Major Changes #3 above, we show explicitly how our method is faster than that of Corces. In our method, we do not need to perform reads count for merged targeted regions. With the above points in mind, we can conclude that our method is simpler.

Comment B-3)

What does the color scale mean in Figure 1?

Reply B-3)

The color scale indicates the number of data in the bin, which is explained in the caption of Figure 2 in the revised manuscript.

Comment B-4)

Lambda is hard to interpret across different runs and datasets. The authors may consider using a standard cluster purity measure, which ranges from 0 to 1

Reply B-4)

As mentioned in Major Changes #2, it is difficult to use the concept of cluster purity. Thus, we needed to introduce another quantity to evaluate the dendrograms, for which we used the penalty. Note that the penalty could be normalized from 0 to 1 by dividing the maximum value 924 if needed.

Comment B-5)

Section "Robustness of the clustering" can be further subdivided into two sections (clustering methods and downsampling) to improve readability.

Reply B-5)

We subdivided the section into two sections, as suggested.

Comment B-6)

What do different colors mean in Figure 13?

Reply B-6)

We have revised the figure to so that each circle indicates one sample and each square indicates the average over samples at the same r value.

Comment B-7)

Supplementary Section V says that the cost of the optimization algorithm depends quadratically on the length of the DNA sequence (L_0). However, it is unclear why this is the case since computing the Hamming distance between two binary strings takes linear time in their length.

Reply B-7)

We thank the reviewer for this comment. The point by the referee is true, and our statement was a mistake. The text has been revised accordingly.

Comment B-8)

Supplementary Section V: it is not clear what the constants K and K' mean?

Reply B-8)

Importantly, the constants do not depend on the data size or our optimization resolution. However, since they were confusing, we use the Landau notation of O() in the revised manuscript.

Comment B-9)

The description of the algorithm should state more clear whether the genome is binarized at 1bp or at a more coarse resolution

Reply B-9)

The genome is binarized at 1 bp. We insert this statement at the end of section "Parametrized binarization" in the revised manuscript.

Comment B-10)

The manuscript should state whether MACS2 was actually run with all the possible values for the p-value threshold (P_G). The text in Sup. Section V appears to downplay a bit this part, but running MACS2 for many values of P_G would take long time in practice.

Reply B-10)

It is impossible to run all possible values for p_G because it takes any positive real number. Our strategy is to look for a value close to the optimized value of p_G by discretizing p_G. One can decide the searching region in the discretized space of p_G depending on the computational resources.

We thank the referee for all the insightful comments that have helped us improve our paper.

Reviewer #2 (Evidence, reproducibility and clarity (Required)):

In Tanaka et al., the authors create a new method of data analysis for accessible chromatin data with application of their new strategy to ATAC-seq samples from the hematopoietic system. They use a new genome-based hamming distance metric to infer properties of samples, such as clustering cell types and classifying out-of-sample libraries, including those derived from patients with AML. The manuscript is generally well-written with extensive use of equations and variables that is appropriate for a bioinformatics audience with expertise in mathematics or computer science.

Reviewer #2 (Significance (Required)):

A new method for ATAC-seq analysis. Ideally should compare with existing methods, as suggested.

Unclear if this is better than existing methods and therefore benchmarking is needed.

Unclear audience, but should better define with revision. Ideally would be revised for computational journal, but need to improve benchmarking and innovation for computational audience.

Our expertise: Epigenomics, hematopoiesis, and computational biology

****Major****

Comment C-1)

The premise of the manuscript is to present a new method. However, the authors make effectively no effort to benchmark their approach against other methods, most notably those found in the Corces, et al. 2016 work. Do the authors have any proof that their approach works any better than those used previously with these data? Specifically, does the classification of new cell types using the author's methods work better than the use of enhancer cytometry (via a support vector machine regression)?

Reply C-1)

As explained in Major Changes #2 in the top of this letter, our best dendrogram is no worse than the dendrogram in Figure 2f of Corces et al.

Comment C-2)

There has been considerable noise at the base-pair level with ATAC-seq data whether called as a peak or not. Perhaps unsurprisingly, the results shown in the hierarchical fail to perfectly segregate cell types (even in Figure 10 which apparently has optimal results). If anything, these results suggest that the authors methods underperform relative to other approaches, and further emphasize the need to bench mark against other approaches.

Reply C-2)

As explained in Major Changes #1 in the top of this letter, our performance of the clustering is improved when taking into account the parameter space of M_cut with Ward's method. At this time, we are unaware of any methods that provide better clustering without simply increasing the resolution of our searching parameter.

Comment C-3)

While other methods may rely upon certain assumptions that the authors try to avoid (as motivated in the introduction), this approach also has its drawbacks. Specifically, the run time cost of computing a hamming distance at the base-pair resolution limits its utility to other data, such as single-cell ATAC-seq.

Reply C-3)

We thank the reviewer for this pertinent comment. If we regard one read of single-cell ATAC-seq as one peak region in our study, the computational cost to calculate Hamming distance is the order of total reads number. Then, the applicability of Hamming distance from the viewpoint of its running time depends on the total reads number of a given single-cell ATAC-seq. We would like to develop our current algorithm for application to single-cell ATAC-seq in future study.

Comment C-4)

The audience that would find this paper accessible and interesting is unclear, due to the the presentation of the work. On one hand, it is inaccessible to most biologists as written. On the

other hand, there are limited innovations presented that would be of interest to a bioinformatics audience. The authors should consider revising their presentation for a specific audience.

Reply C-4)

In the revised version of our manuscript, we reanalyze the data set by Ward's method to improve the clustering compared to the original version. Further, we clearly state how our algorithm is faster than the standard methods such as Corces et al. In that sense, the updated content in the revised version includes enough innovations that should be presumably of interest to a bioinformatics audience. Further, we apply our algorithm to two more types of leukemia, CLL and ATL to assess the applicability of our method. From those viewpoints, we believe that our updated work could be presumably of interest to both biologists specializing in cancer studies and bioinformaticians specialized in genomic analysis.

****Minor****

Comment D-1)

The inclusion and choice of figures for the manuscript seems superfluous. It's not clear what benefit, if any, several figures bring to describing the method. For example, Figures 9 and 10 are not referenced in the text.

Reply D-1)

In accordance with the referee comments, Figures 9 and 10 were superfluous and removed from the revised manuscript.

Comment D-2)

Laborious descriptions of common bioinformatics methods such as UPGMA and Hamming have been described extensively elsewhere. The authors' inclusion of descriptions of these methods are not necessary and further convolute an already lengthy manuscript.

Reply D-2)

Our objective is to make this paper as self-consistent as possible. In particular, in order to define the penalty Lambda, we need to explain the clustering method in detail. Though the referee's feeling about lengthy manuscript is understandable, we have decided to keep our style.

We thank the referee for all the insightful comments that have helped us improve our paper.
