# Supplementary material for: Systematic clustering algorithm for chromatin accessibility data and its application to hematopoietic cells
Source: PLoS Comput Biol. 2020 Nov 30;16(11):e1008422. doi: 10.1371/journal.pcbi.1008422 (PMC7728210; doi:10.1371/journal.pcbi.1008422)
Supplement: Attachment — Submitted filename: ReplytoReviewer20200807.pdf [file pcbi.1008422.s003.pdf]

Dear reviewers,

Thank you for giving us the opportunity to strengthen our manuscript with your valuable comments and queries. Following those comments, we have revised our manuscript, which we submit with this file.

Thank you once again for your consideration of our paper.

Sincerely,

Azusa Tanaka, Yasuhiro Ishitsuka, Hiroki Ohta, Akihiro Fujimoto, Jun-ichirou Yasunaga, and Masao Matsuoka

****

Reviewer's Responses to Questions

Reviewer #1: Dear Colleagues,

I read with interest your manuscript on identifying cell or tissue type using ATAC-Seq. In it you present a simple algorithm to compute a distance between samples. The text itself is very well written.

Following on the comments in the previous round of reviews, I have two major questions:

Reviewer #1: Major comments:
Comment 1-1)

What exactly is the advance over prior art? While it is true that there are few ATAC-Seq specific differential analysis pipelines, a number of ChIP-Seq differential analysis tools such as HOMER, DBChIP and DiffBind are evidently commonly used (1). In fact, a quick search reveals that there are roughly a dozen such methods (2) that can be used to cluster samples

based on ChIP-Seq measurements. Have you considered benchmarking your approach against these tools?

Reply 1-1)

We thank the referee for the comments. We believe that our work has two key advances over the previous work by [Corces et al., Nature Genetics 2016]. (1) It provides an algorithm with extremely low computational cost to perform clustering in terms of cell types using ATAC-seq data and surface markers of cell samples, as mainly described in the section "Computational cost of the algorithm". (2) Second, it can infer information about proteins appearing on the surface of hematopoietic cells and leukemic cells by using only information at the chromatin level, which is quantified by ATAC-seq data, as mainly described in the section "Application to leukemic cells". Thus, we are able to obtain elusive information about ATL samples without RNA-seq data.

As for benchmarking, we tried to perform a clustering of 77 samples by DiffBind with default settings, however, the required computational memory capacity was too demanding. Therefore, we focused on only B, NK, CD4+ T, CD8+ T cell samples (N_s=20), because they are all lymphoid cells. For this case, DiffBind with default settings gives a penalty=14 for UPGMA and penalty=11 for Ward's method. Corces' method gives a penalty=14 for both methods. On the other hand, our algorithm gives a penalty=5 with Ward's method. These findings support the superiority of our algorithm. As the referee pointed out, further benchmarking for more refined test data remains to be studied in the future.

Comment 1-2)

Why is the speed of calculation an issue? Are existing methods too slow? Even assuming a large number of datasets to be compared, computing a matrix of pairwise comparisons is embarrassingly parallel hence the term N_s is not necessarily a concern in practice.

Reply 1-2)

We agree with the referee that the "speed" depends on the amount of parallel computation. However, in reality, there is a limitation for computational power. We have shown that the computational cost of our algorithm is much less than that of Corces' in the section "Computational cost of the algorithm. Especially, when the computational power is limited, the difference of order in the cost between Corces' method and our method in terms of N_s for sufficiently large N_s could be significant. In the future, as ATAC-seq datasets become larger, the advantage of our algorithm will become more evident even when using parallel computation. Moreover, one of the current focuses in the field of ATAC-seq is single-cell ATAC-seq. When we consider single-cell ATAC-seq data, typically we have to deal with N_s = 10000 [Cusanovich et al., Science 2015, Cusanovich et al., Nature 2018, González-Blas

et al., Mol Syst Biol 2020]. Thus, from the viewpoint of computational cost, our current algorithm could be a basis for new algorithms for the analysis of single-cell ATAC-seq.

Minor points:

Comment 1-3)

l85. What does the length of the peaks have to do with data reduction? If you store each peak as a triple (¥gamma, ¥alpha, ¥beta) (as in l.73), then the difference between ¥beta and ¥alpha has no incidence on information content.

Reply 1-3)

Thank you for your comment. Figure 2 shows that when we define the length of peaks as ¥beta-¥alpha, the lengths shorten as the associated p-value rises. Because of this relationship, truncating the set of peaks with larger values of M_cut is expected to improve the robustness against small noise in the data.

Revision regarding 1-3）

line 102: We have added comment about robustness and noise in the section “Parametrized binarization” in Results.

Comment 1-4)

l131. Do you have evidence that you algorithm is significantly faster than the approach described in Corces et al? A difference in computational complexity (as derived below) is not a proof of speed, just asymptotic behaviour.

Reply 1-4)

We appreciate the referee’s comment. For this issue, we calculated the speed of our clustering method for N_s=77 and confirmed that our algorithm was significantly faster in practice. For this calculation, we considered a situation where we had already calculated the distances between 76 samples and would like to add one more sample (in our trial, it was B cells) which had not been analyzed. Note that for simplicity, we do not consider the time to perform the peak calling, because both the method by Corces and our algorithm need this process. This process adds about 5 minutes to both methods. Thus, the peak-calling process does not affect the argument below.

When we used Corces' method, (i) counting reads in each targeted region in the whole genome took a few minutes, and (ii) calculating Pearson correlation coefficients between 77 samples took about 150 s. Additionally, before (i), the reads data had to be merged (merge bam files), which took at least 30 minutes (iii).

On the other hand, in our algorithm, we don't have to merge the reads data (iii) nor count the reads (i). We only have to calculate distances between newly appearing pairs of samples (ii), which took only 2 s. Thus, even looking only at process (ii), our algorithm is 150/2=75 times faster. If we take into account processes (i) and (iii), the difference is more than that.

For both cases, to calculate all unknown distance values between sample pairs, we used parallel computation. Thus, the comparison mentioned above is consistent with our estimate described in the section "Computational cost of the algorithm".

Comment 1-5)

l151. I don't understand what the comparison of N_r and L_1 to M*_cut brings to the complexity analysis. The Landau notation is about the trend of a function, and the constants within the O(...) are have no bearing to the actual run time, nor to the asymptotic behaviour.

Reply1-5)

Our intended statement is that N_r, L_1 and M*_cut contribute linearly to the speed of Corces' algorithm and our algorithm, respectively, for large values. We do not have information about the actual coefficients to exactly estimate the computational cost for both algorithms except for the Landau notation. Note that because of practical computation speed, we expect that the actual coefficients for the two algorithms are not very different each other.

Revision regarding 1-5)

lines 165-203: Please refer the section "Computational cost of the algorithm".

Comment 1-6)

l173. Are you applying the same M_cut to all samples? Would this not affect results on cell types that have significantly more/fewer accessible regions than others?

Reply1-6)

Yes, we have applied the same M_cut to all samples. Indeed, the total length of the regions cut by M_cut depends on each sample. In our approach, this is not a problem. We rather

regard this fact as a natural consequence of analyzing different cell types. Our approach is to find a potentially robust M_cut and apply it to unknown samples such as leukemia.

Revision regarding 1-6)

line 274: We inserted “with optimized parameters (M_cut*, p_G*)”.
line 305: We inserted “(M_cut*, p_G*)=(64000,10^-2) is used”.

Once again, we appreciate you taking the time to offer us your insightful comments on our paper.

Reviewer #2: Major comments:

Comment 2-1)

Some inconsistent concepts are being used throughout the manuscript. In the title, the authors claimed it's a "clustering algorithm". However, in the abstract and the main text, the authors descripted it as a 'data reduction method'. And meanwhile, it was explained as a 'classification method'. These concepts are all essentially different and mixing them up makes this manuscript read confusing. Especially 'clustering algorithm' and 'classification' are self-contradictory. Apparently, the authors were utilizing known labels to optimize the loss function or classify samples so it should not be considered as an unsupervised clustering method. The authors need to be careful, consistent, and clear about the method description.

Reply 2-1)

We appreciate the referee's comment. Our algorithm consists of three steps: (i) clustering, (ii) optimization, and (iii) classification.

(i) The clustering step gives a dendrogram from ATAC-seq data. As input, we need to choose two parameters, (p_G, M_cut), and the method of hierarchical clustering, e.g. UPGMA or Ward's method.

(ii) For the optimization step, we assume that the samples are classified into cell types and choose a hierarchical clustering method. To measure how the obtained dendrogram fits into the pre-defined cell-type classification, we introduce a "penalty". Then we seek and use (p_G*, M_cut*) with the lowest penalty.

(iii) For the classification step, we assume that new ATAC-seq data which are not used in the previous two steps are given. Using (p_G*, M_cut*) from (ii), we produce a dendrogram from the supervising samples and one of the new samples. By considering which cell-type is closest to the new sample in the obtained dendrogram, we can classify the new sample.

With these explanations, we intend the following meanings for the ambiguous terms.

- "Clustering algorithm" , which is equal to "systematic clustering algorithm" refers to step (i).
- "Classification" refers to step (iii), but does not specify the algorithm.
- "Data reduction method" refers to the binarization of the peaks data in step (i) and is described in the section "Parametrized Binarization".
- "Hierarchical clustering methods" which is used only in line 229 is the method we used in step (i).

In the revised manuscript, we clarify the relation between "clustering algorithm" and "data reduction" in the Abstract and Introduction.

Revision regarding 2-1)

lines 15-18 and 64-65: We modified sentences to clarify the relation between "clustering algorithm" and "data reduction method".

line 127: We changed "for cell-type classification" to "toward cell-type classification" (we corrected the preposition).

lines139: We changed "for the best cell-type classification" to "for the optimization".

lines 235, 237: We changed "the classification result" to "type penalty" to clarify by what quantity we estimate our classification.

line 342: We changed "systematically classify" to "systematically perform clustering of".

line 344: We changed "for the best cell-type classification" to "toward cell-type classification".

lines 447, 448: We changed "for better cell classification" to "for the cell-type classification".

Comment 2-2)

For the parameter optimization, instead of using grid search, the authors narrowed down the range of PG first by setting Mcut to infinity and then determine the best parameter pair within the range. But this can be problematic since both parameters contribute to the loss function simultaneously. In other words, the optimal Mcut might fall outside of the range of PG [1.5,4] so the best parameters determined this way might not be optimal, especially when no clear correlation between PG and Mcut was not observed in Figure9 and Figure 10. The authors need to further justify the parameter optimization.

Reply 2-2)

Thank you for the comment. Our optimization protocol can be regarded as a grid search of -log p_G with [1.5,4] and M_cut with [1000,100000], though the searching points are heterogeneous in parameter space (p_G, M_cut). We used the term "best parameters" for the values that achieve the minimum penalty in our searching resolution of the parameter space. Note that these "best" parameters are generally different from the values in parameter space (p_G, M_cut) that truly optimize the penalty. Our argument is not that the computed

best parameters (p*_G M*_cut) are the true optimal. Indeed, finding the true optimal in optimization problems for a two-dimensional space, where the axis of p_G is positive real numbers, is generally unsolvable. Nevertheless, as we search for the parameter space in a more fine-grained manner, we can find better parameters in terms of the optimization.

Revision regarding 2-2)

lines 150-152: We added “Note that more-refined resolutions might give better estimates of the optimized value (M_cut*,p_G*), but naturally the computational costs get higher. Even then, the following procedures are operationally unchanged.”

Comment 2-3)

Can the determined ‘best parameters’ be generalizable to other cases? It seems different linkage criteria may already result in different solutions of parameters, which means the parameter optimization is a bit sensitive. How to decide the parameters when no labels are given? Also, in the application to leukemic cells, how were the parameters determined?

Reply 2-3)

In order to determine candidate optimized parameters (M*_cut, p*_G), we need the labels of all samples. After these candidates are determined by clustering the 77 samples, we fix (M*_cut, p*_G) and analyze leukemia samples accordingly. In this sense, we assume that (M*_cut, p*_G) are valid to characterize new samples such as leukemia samples. This process is standard in supervised learning. On this assumption, after fixing (M*_cut, p*_G) , we do not need labels to compute which cell type is closer. What condition allows the assumption to hold is for future study.

Revision regarding 2-3)

line 274: We added “with the optimized parameters (M_cut*, p_G*)”

line 305: We added “(M_cut*, p_G*)=(64000,10^-2) is used”

Comment 2-4)

The authors claimed the proposed algorithm works better than quantile normalization however this manuscript lacks a stringent comparison between these two strategies. Line 166-167, although the authors did a basic comparation, comparison details are not described. Are both methods using the same regions? Do the clustering solutions have the same number of clusters? Does the linkage criterion ‘Ward’ give the best performance for quantile

normalization? Instead of hierarchical clustering, will another clustering method suit quantile normalization better?

Reply 2-4)

Thank you for the comment. The previous algorithm uses a merging method for peaks to define targeted regions, whereas our algorithm's target is the whole genome region. Therefore, the regions contributing to the distance matrix are different. For the merging method by Corces, Ward's method gives a better penalty=27 than UPGMA penalty=29. For non-hierarchical clustering methods, we do not know how to determine the cluster number, which requires an additional assumption. In our algorithm, we do not assume the number of clusters in the clustering and thus we do not consider such cases. As the referee pointed out, our results do not deny the possibility of a better method combined with quantile normalization.

Revision regarding 2-4)

lines 159,160: We added "Note that for this case, Ward's method gives penalty lambda =27 and UPGAM gives lambda =29."

Comment 2-5)

How to determine the best parameters for cell-type classification is a critical part for the proposed algorithm. That being so, it makes more sense to put the section of 'Computational cost of the algorithm' after "Determination of the best parameters in the optimization".

Reply 2-5)

In accordance with the referee's comment, we switch the order of the two sections in the revised manuscript.

Comment 2-6)

Instead of overstating the method as a systematic clustering algorithm for 'epigenetic data', it would be better to be more specific since in this manuscript, the algorithm is mainly designed for ATAC-seq and has only been tested on ATAC-seq data.

Reply 2-6)

We have modified the title as the referee suggested.

Revision regarding 2-6)

We changed the title "Systematic clustering algorithm for epigenetic data and its application to hematopoietic cells" to "Systematic clustering algorithm for chromatin accessibility data and its application to hematopoietic cells".

Comment 2-7)

The authors should consider re-organizing the figures and tables (E.g. There is no need to separate table2-4. They can all be merged into one table) to highlight the primary results and improve the readability. Many of them can go to supplementary figures. It would also be very helpful if the authors can add a main figure to illustrate the workflow of the proposed algorithm.

Reply 2-7)

Following the referee's comment, we have reorganized Tables 2-4. We keep the structure of the sections because in our opinion, it is very important for this paper. We have added the workflow as new Figure 8, as the referee suggested.

Revision regarding 2-7)

We reorganized Tables 2-4 into Table 2 in the section "Application to leukemic cells".

We added new Figure 8 in the section "Determination of the best parameters for the optimization" and explained on line141.

Minor comments:

Comment 2-8)

Please define the superscript 'c' in Figure3 when first introducing it.

Reply 2-8)

We thank the reviewer for this comment. Following the comment, we have added an explanation of 'c' in the caption of Figure3.

Comment 2-9)

Figure7 legend. The correct term for ‘broken line’ is ‘dashed line’

Reply 2-9)

We have modified Figure 7 legend as suggested.

Comment 2-10)

Line 164 “Our searching resolution in terms of increasing M_cut was 1000 near M_cut = 64000.” What is ‘1000’ here?

Reply2 -10)

We apologize. 1000 was an error. The number should have read 2000.

Revision regarding 2-10)

lines 149-152: We have revised the manuscript accordingly.

Comment 2-11)

Line 287 ‘substantial overlap’. Please give the specific overlap ratio.

Reply 2-11)

We added the overlap ratio for pHSC and blast, but excluded LSC for the reason explained in the main text.

Revision regarding 2-11)

lines 321-323: We added “where 12 out of 17 samples for pHSC and 13 out of 18 samples for Blast are overlapped”.

We would like to again thank the referee for all the insightful comments that have helped us improve the paper.
