# Supplementary material for: Systematic clustering algorithm for chromatin accessibility data and its application to hematopoietic cells
Source: PLoS Comput Biol. 2020 Nov 30;16(11):e1008422. doi: 10.1371/journal.pcbi.1008422 (PMC7728210; doi:10.1371/journal.pcbi.1008422)
Supplement: Attachment — Submitted filename: response_to_reviewer_200905.pdf [file pcbi.1008422.s004.pdf]

Dear reviewer,

Thank you very much for providing us the constructive feedback regarding our manuscript. Following the comments, we hereby resubmit our revised manuscript.
Thank you once again for your consideration of our paper.

Sincerely,
Azusa Tanaka, Yasuhiro Ishitsuka, Hiroki Ohta, Akihiro Fujimoto, Jun-ichirou Yasunaga, and Masao Matsuoka

****

Reviewer #1: Dear Colleagues,

Comment)

I still do not understand however why your comparison to prior art consists of only one method. Further, the selected competitor was not particularly advanced: the Corces 2016 paper was essentially focused on a new and exciting dataset, not a refined analysis method. I take on board that DiffBind was too slow, but as pointed in my previous review, there are at least a dozen other methods out there.

People have long been doing epigenomic similarity matrices on ChIP-Seq and DNAse-Seq. For example, the website of the international human epigenome consortium (IHEC) allows you to compute correlation matrices on all their datasets. Also, the CODEX project (1) has been using the Dice coefficient, which is very similar to the Hamming distance used in your method. Why can't these existing methods simply be used on ATAC-Seq

data?

(1) https://academic.oup.com/nar/article/43/D1/D1117/2439489

Reply)

Thank you for the comment about extending our comparison with other methods. In our current paper, the main focus is to find a method that improves cell type classification compared to Corces' 2016 paper and to apply the new method to other cell samples such as unanalyzed leukemia cells. In this sense, the term "standard method" that we used in the previous draft is an overstatement. Thus, we have revised our manuscript as explained below. As the reviewer pointed out, we should analyze more samples to check if our method is robust.

Also in accordance with the reviewer's comment, we computed the Dice coefficient for several values of M_cut with $p\_G=10^{-2}$. As seen in the figure below, the Dice coefficient with Ward's method gave 29 as the minimum value of penalty, whereas our method gave a value of 18. Although the reason for why the Hamming distance gives a better result compared to the Dice coefficient is not clear, this observation implies that the width of each peak affects or reflects the cell phenotype, because the Dice coefficient tends to neglect the effects of the width. We plan more comparisons with other existing methods and data in the future.

Figure

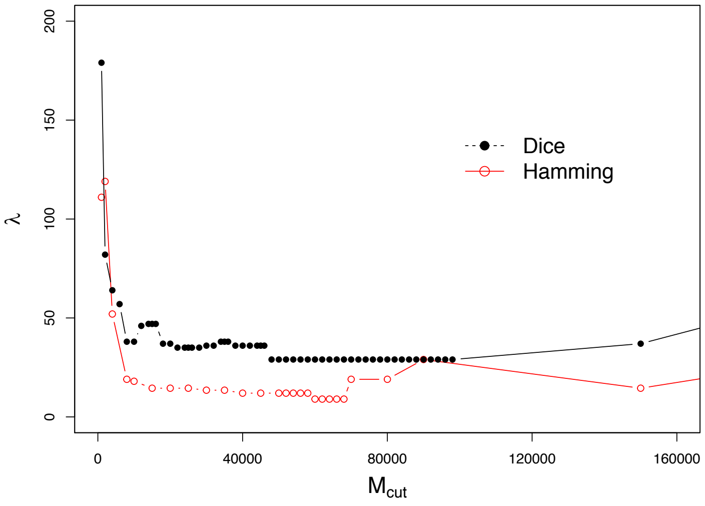

Global penalty calculated by the Hamming distance and Dice coefficient as a function of M_cut with p_G=10^{-2}.

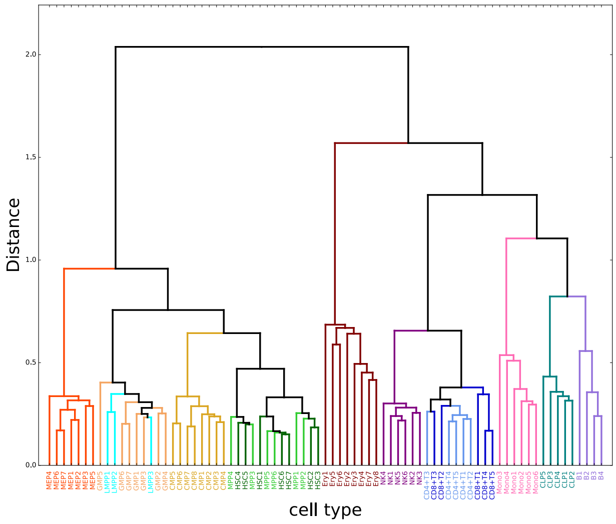

A representative result of the clustering completed by using the Dice coefficient with Ward's method.

(M_cut = 64000 with p_G=10^{-2})

A summary of the revisions to the manuscript are given below.

line 33: “standard methods” was changed to “a previous method (Corces et al, 2016)”

line 67: We added “compared to a previous method (Corces et al, 2016)”.

lines 172, 189, 199, 346, 350: “standard methods” was changed to “a previous method”

line 355-361: We inserted the following paragraph:

As a next step, we need to investigate if constructed algorithm is robust for other existing methods and data. For example, for the same data of hematopoietic cells, we replaced the Hamming distance with the Dice coefficient, which has been used in the CODEX project (Sánchez-Castillo et al., 2015) to quantify differences between two samples but found the results with p_G=10^-2 were not improved in terms of the penalty. We also compared our algorithm with DiffBind (Ross-Innes et al., 2012), which is commonly used as a ChIP-seq differential analysis tool, but again found that DiffBind with its default setting did not give a better clustering result. Note that there are other existing methods and data to be checked in the future.

line 376: We added “available, for example, from The International Human Epigenome Consortium (https://epigenomesportal.ca/ihec/), ROADMAP Epigenomics (http://www.roadmapepigenomics.org/) and many other resources” for better explanation of the data accessibility.

We would like to again thank the referee for the valuable comments that have helped us improve the paper.
